# Genetic biomarkers and crucial cell subsets of iron metabolism in Beta-Thalassemia: insights from bioinformatics and experimental validation

**DOI:** 10.1007/s00277-025-06605-6

**Published:** 2025-09-16

**Authors:** Renrong Wei, Dan Qiu, Xiangxing Zeng

**Affiliations:** 1 Medical laboratory, Heyuan Key Laboratory of Molecular Diagnosis & Disease Prevention and Treatment, Doctors Station of Guangdong province, Heyuan People’s Hospital, No.733 Wenxiang Road, Yuancheng District, Heyuan, Guangdong China; 2 Department of Traditional Chinese Medicine, Heyuan People’s Hospital, Heyuan, Guangdong China

**Keywords:** Thalassemia, beta, Iron metabolism, Biomarkers, genetic, Erythrocytes, Gene expression profiling, Animal models

## Abstract

Approximately 1.5% of individuals with hemoglobin disorders carry the β-thalassemia gene variant, impacting around 40,000 newborns annually. Given the incomplete understanding of β-thalassemia pathogenesis, there is an urgent need to identify effective biomarkers to advance research, diagnosis, and treatment. This study aims to identify potential biomarkers for two key purposes: (1) diagnosing transfusion-dependent β-thalassemia (TDT) and (2) detecting iron overload complications, with a focus on functional markers that reflect iron metabolism dysregulation in TDT. This study integrates transcriptomic data from the Genome Sequence Archive dataset (CRA003639) with bioinformatics analysis to identify potential biomarkers associated with β-thalassemia. Subsequently, Hbb-bs and Hbb-bt double knockout mice were used to establish a β-thalassemia model, while C57BL/6JCya mice served as the control group, to validate the identified biomarkers through animal experiments. Seventeen reliable cell subsets were identified through rigorous annotation and screening. Quantitative analysis revealed a decreased proportion of immune cells (natural killer [NK] cells, T cells, macrophages, neutrophils, and monocytes) and an increased proportion of erythroid cells in the β-thalassemia group. Cell subset analysis focused on subsets that closely communicated with erythroid cells. Enrichment analysis of driver genes in these subsets revealed iron metabolism–related pathways in *Erythroid_02* and *Erythroid_03*, and a ferroptosis-related pathway in *Erythroid_05.* Thalassemia model mice exhibited stronger iron ion fluorescence signals in primary hepatocytes, increased levels of total iron, Fe^2+^, and Fe^3+^ in liver tissue, and decreased serum iron (SI) levels, indicating iron metabolism disorders. Reverse transcription polymerase chain reaction (RT-PCR) results showed differential gene expression, with *BCL2L1*,* Hepb1*, and *Prdx6* downregulated and *Spta1* and *Snca* upregulated in the TDT model group. This study comprehensively characterizes TDT at the cellular and molecular levels, offering insights into its pathogenesis and identifying potential therapeutic targets.

**Supplementary Information:**

The online version contains supplementary material available at 10.1007/s00277-025-06605-6.

## Introduction

Thalassemia comprises a group of inherited hemolytic anemias caused by partial or complete impairment in the synthesis of α- or β-globin chains. In southern China (primarily regions south of the Yangtze River), the incidence of thalassemia is relatively high and is associated with increased mortality. It represents the most common cause of secondary iron overload [1]. In these areas, β-thalassemia is particularly prevalent and tends to exhibit more severe genotypes than α-thalassemia. Based on gene mutations and clinical presentation, β-thalassemia can be classified into thalassemia minor (caused by a single gene mutation) and homozygous double gene mutations, including β-thalassemia major and β-thalassemia intermedia. Heterozygous forms also exist, such as sickle cell β-thalassemia and hemoglobin E/β-thalassemia. Currently, β-thalassemia is categorized into two main types: transfusion-dependent and non-transfusion-dependent [2]. In severe forms like β-thalassemia major, regular blood transfusions are the standard treatment to maintain adequate hemoglobin levels [3]. However, repeated blood transfusions and increased intestinal iron absorption can lead to iron accumulation in the major organs, which in turn causes cardiac and liver dysfunction [4]. If aggressive iron chelation therapy is initiated as early as possible, iron-related organ dysfunction may be reversed [5].

Gene expression analysis methods based on transcriptomes have been widely used in biomedical and clinical research in recent years and are particularly suitable for identifying disease-related biomarkers [6]. This method can help researchers detect differentially expressed genes that may serve as potential biomarkers for diagnosis, prognosis, and treatment response. In the study of β-thalassemia, transcriptome analysis has been used to explore its molecular mechanisms. A recent study investigated the immune characteristics of peripheral blood mononuclear cells of patients with thalassemia through transcriptome analysis and found that natural killer (NK) cell-mediated eosinophil chemotaxis could activate reactive oxygen species (ROS), which are associated with disease severity. In addition, transcriptome analysis has shown that the expression of the ABO gene is significantly increased in patients with severe thalassemia, which might enhance the immune response and increase the demand for blood transfusion [7]. Another study conducted transcriptome analysis on β-thalassemia patients and found that the differentially expressed genes were significantly enriched in pathways related to β-thalassemia, including hematopoiesis, heme biosynthesis, oxidative stress response, inflammatory response, immune response, circadian rhythm regulation, apoptosis, and other cellular activities [8]. In this study, we integrated recently published transcriptome data of β-thalassemia and screened for biomarkers related to β-thalassemia by combining multiple bioinformatics methods, and then performed functional enrichment analysis on these genes. Subsequently, these biomarkers were verified through animal experiments to reveal the key cell subsets involved in iron metabolism in β-thalassemia and their driving genes. The term ‘biomarker’ refers to genes or molecular indicators whose expression patterns correlate with transfusion-dependent β-thalassemia (TDT) pathogenesis and iron overload complications. Specifically, we aim to identify: (i) diagnostic biomarkers that distinguish TDT from normal states at the transcriptional level; and (ii) functional biomarkers associated with iron metabolism disorders, which may indicate the risk or severity of iron overload in TDT.

## Materials and methods

### Bioinformatics analysis

#### Data sources

In this study, we initially screened the GSE133181 dataset from the Gene Expression Omnibus database, which included samples from patients with TDT and healthy controls. Through data quality control, we found that in the pre-sequencing process of the original study, CD34 + hematopoietic stem cells were retained via cell sorting. However, a highly undifferentiated cell population does not effectively reflect the iron metabolism characteristics of mature blood cells, making it difficult to directly analyze the association between the pathological mechanisms of TDT and iron metabolism. Therefore, we switched to the alternative dataset CRA003639 from the Genome Sequence Archive database for analysis. This dataset contains bone marrow samples from four TDT mouse models and four wild-type mice. In the absence of direct evidence of association at the single-cell level, the research strategy was adjusted to conduct gene set enrichment analysis using the Molecular Signatures Database and to establish an iron metabolism-related regulatory network by integrating the Human Metabolome Database. To align with our objective of identifying iron overload-related biomarkers, bioinformatics analyses prioritized driver genes enriched in iron metabolism and ferroptosis pathways, ensuring candidate biomarkers were functionally relevant to iron overload complications.

#### Quality control and cell dimensionality reduction

FASTQ data are the raw output of high-throughput sequencing. For data analysis and quality control, we used the CellRanger software suite. Due to the large number of cells in this dataset, which made cell clustering difficult, we added a doublet removal step after data standardization and filtering. This step was performed using the DoubletFinder package (Figure S1). We employed a Uniform Manifold Approximation and Projection (UMAP) method for data dimensionality reduction, based on the theoretical framework of Riemannian geometry and algebraic topology [9]. UMAP not only has a fast running speed and low memory usage, but also has a significant advantage when dealing with single-cell data; namely, it can reflect the continuity and organization of differentiation among cell populations. After importing the sequencing files processed by CellRanger into Seurat and completing data quality control and filtering, the UMAP method was used to reduce the dimensionality of the data.

#### Cell clustering and annotation

Cell annotation was performed using the automatic annotation R package SingleR, which retrieves marker genes from reference datasets and literature reviews, and refers to relevant analyses conducted using the clusterProfiler package. Automated annotation using SingleR typically utilizes precompiled reference datasets, and the operation is relatively straightforward. This software annotates cell subsets with specific cell types by importing the cell clustering results into SingleR. When conducting manual annotation, we combined the annotation results from SingleR with references from the literature and the CellMarker database [10]. The cell subsets were annotated based on the average expression levels of marker genes and their expression proportions within the cell subsets.

#### RNA velocity

RNA velocity leverages the fact that newly transcribed unspliced precursor messenger RNAs (mRNAs) and mature spliced mRNAs can be distinguished in common single-cell RNA sequencing workflows to recover directional and dynamic information about gene expression [11]. scVelo addresses the complete transcriptional kinetics problem using a likelihood-based kinetic model. Moreover, scVelo can infer gene-specific rates of transcription, splicing, and degradation, and recover the latent time of cellular processes [12]. scVelo was used to analyze RNA velocity in this study.

#### Cell communication

Cell communication serves as the foundation for multicellular organisms to coordinate various physiological processes. It enables cells to collaborate and regulate each other, thereby maintaining the normal growth, development, metabolism, and homeostasis of the organism. Based on the gene expression characteristics of different cell populations, cell communication analysis can identify specific ligand-receptor pairs between cell types, reflecting their communication status. We used the CellChat package to conduct the cell communication analysis. CellChat integrates cell clustering information and calculates communication probabilities at the signaling pathway level by aggregating the interaction probabilities of ligand-receptor pairs associated with each pathway. After inferring the intercellular communication network, to further explore and analyze the data, we used CellChat to identify and analyze the specific signaling pathways involved in cell communication.

#### Cell subset analysis

The Seurat package was used to extract specific cell subsets for in-depth analyses. We performed dimensionality reduction on these extracted subsets and used scVelo to analyze their RNA velocity. We estimated cell-to-cell transition probabilities to project the velocity onto a low-dimensional embedding. In other words, for each velocity vector, we identified possible cell transitions aligned with the vector’s direction. Subsequently, we conducted latent and pseudotime analyses on the cell subsets of interest. We then determined the role of these subsets in the disease based on their degree of differentiation and extracted the genes contributing most to this role for enrichment pathway analysis to explore molecular mechanisms during disease progression. Additionally, we extracted cell subsets at key differentiation nodes and performed RNA velocity, latent time, and pseudotime analyses. scVelo calculates the probability of each gene and cell given the optimal latent time and transcriptional state, thereby evaluating how well the learned spliced/unspliced RNA phase trajectories describe cell states. By aggregating the cells, we obtained the overall gene probabilities and ranked the genes according to their goodness-of-fit to identify genes exhibiting distinct dynamic behaviors. These genes may be important drivers of key processes in cell populations. We ranked the driving genes calculated using scVelo based on splicing kinetics according to their goodness-of-fit and selected the top 50 genes for enrichment analysis.

A detailed workflow of the transcriptomic data analysis pipeline, including data preprocessing, quality control, dimensionality reduction, cell clustering, annotation, and downstream functional analyses, is provided in Figure S2.

### Experimental validation

#### Animals and experimental design

The animals were divided into the model and control groups. The model group consisted of five Hbb-bs and Hbb-bt double knockout mice, while the control group included five C57BL/6JCya mice. Subsequently, pentobarbital sodium (50 mg/kg) was administered via intraperitoneal injection. After anesthesia, blood samples were collected by enucleation. The samples were transferred to Eppendorf tubes and allowed to stand at room temperature for 30 min. The samples were then centrifuged at 3000 rpm for 15 min. The serum supernatant was carefully aspirated and transferred to new EP tubes for later use.

#### Biochemical indicators in serum and liver tissue

According to the kit instructions, the following methods were used for detection: (1) serum: serum iron (SI) and ferritin contents were determined by colorimetry; (2) liver tissue: oxidative stress indicators, including ROS level, malondialdehyde content, and activities of superoxide dismutase and glutathione peroxidase, were measured by fluorescence probe method, while iron metabolism indicators (Fe^2+^, Fe^3+^ and total iron content) were detected by chemical colorimetry.

#### Reverse transcription polymerase chain reaction (RT-PCR)

Liver tissue mRNA was extracted using the TRIzol extraction method, and total RNA concentration was measured. The RT-PCR reaction system consisted of 1 µL complementary DNA template, 0.4 µL each of forward and reverse primers, 8.2 µL double-distilled water, and 10 µL 2× SYstemic Biology Reagent Green PCR Master Mix, totaling 20 µL. The amplification reaction conditions were: pre-denaturation at 95 °C for 3 min; followed by 40 cycles of denaturation at 95 °C for 10 s and annealing/extension at 60 °C for 30 s. The 2^-ΔΔCT method was used to calculate relative gene expression levels. The primer sequences for all target genes and the reference gene are listed in Table 1. All primers were designed using Primer-BLAST (NCBI) to ensure specificity, with amplicon lengths ranging from 180 to 250 bp. Melting curve analysis confirmed a single peak for each primer pair, indicating no non-specific amplification or primer dimer formation.

**Table 1:** Primer sequences used for RT-PCR analysis

Gene symbol	Sequence (5’−3’)
Add1	F: GCCTTAAAGAAATGGGTTGCAG
	R: CTGAATGTGGTTTTGGTCATAGG
Atpif1	F: CCTGATTCGCGGTTTCCGTG
	R: TTGTCTTCGGCGTTGCTCAG
Bcl2l11	F: TTCCTAGTTTGAGGTCTGTGG
	R: CCATGAAGTCACATGGCTCT
Eif2ak1	F: GCTGAAGAGATGAGAGGGAAAA
	R: TGTCCTCACTAAGGGTTGGA
Fech	F: ATATGGGCTTAGCTCCCCAG
	R: ATTCAAGGCTATCAGACACAGTC
Hebp1	F: CCTACCAGGGTGATGTCTATTAC
	R: GTTCATCAAGTGACTCATGCC
Hmgb2	F: GACAATGCTGGAGATAGAACCC
	R: CAGGTTTGGGAGAAAATAGCAAAG
Lom2	F: TGAACAAGACATCTACGAGTGG
	R: CCTAAGAGTGAAGACCACACC
Cat	F: TACTCCCAGGTCCTCTTCAA
	R: ATGGCAGAGAGTAGGTTGAC
Slc25a37	F: CTGGTGTCCTATTATCACTGAC
	R: GTGGTATAGGAATAGTTTGGGG
Snca	F: ATGTCATTGCACCCAATCTCC
	R: TCACTGCTGATGGAAGACTTTG
Spta1	F: AGAGGACTCAATTACTATCTGCC
	R: TCTGACTCCTTGTCAATCAGG
Zfpm1	F: ACTAGAGAGAGTGAAACCTTGTTC
	R: AGGCCCAACTCTGTAGTCATA
GAPDH	F: TGAACGGATTTGGCCGTATTG
	R: GTTGATGACAAGCTTCCCATTC
Prdx6	F: CTGGAGAGTTAAATTCAAGGCAG
	R: CCAGACTTCCCATTAAAAGCAG

#### Cell counting Kit-8 assay

The euthanized mice were immersed in 75% alcohol for disinfection. The abdomen of each mouse was then incised in a laminar flow hood to expose the liver tissue. The liver tissues of the mice were cut using sterile instruments, placed in a medium containing phosphate-buffered saline and ethylene glycol-bis(β-aminoethyl ether)-N, N, N’, N’-tetraacetic acid, and cut into small pieces. The minced liver tissue was added to Dulbecco’s Modified Eagle Medium containing collagenase and digested in a 37 °C constant-temperature shaking incubator. After digestion, cells were separated by centrifugation at 1000 rpm for 10 min. The supernatant was discarded, and the cells were washed to remove residual trypsin. The isolated hepatocytes were resuspended in Dulbecco’s Modified Eagle Medium containing 10% fetal bovine serum and inoculated into the corresponding cell culture well plates. The primary cells were cultured in a 37 °C cell incubator, and the medium was replaced regularly.

Primary hepatocytes were inoculated into 96-well plates coated with rat tail collagen and placed in a cell incubator to allow the cells to adhere. After the culture plates were incubated in the dark for 2 h, 10 µL of Cell Counting Kit-8 solution was added to each well. Finally, the absorbance was measured at 450 nm using a microplate reader.

#### FerroOrange fluorescence staining

Primary hepatocytes were seeded into fluorescence-compatible culture plates and cultured in a cell incubator at 37 °C with 5% CO₂ until full cell adherence was achieved. The culture medium was then discarded, and the cells were washed three times with serum-free Dulbecco’s Modified Eagle Medium to remove residual serum components. A 1 µM solution of FerroOrange, a fluorescent probe for detecting intracellular ferrous iron, was added to each well. The plate was incubated in the dark at 37 °C with 5% CO₂ for 30 min to allow proper staining. After incubation, the stained cells were immediately visualized using a fluorescence microscope.

#### Statistical analysis

Statistical data are presented as the mean ± standard deviation. An unpaired two-tailed Student’s t-test was conducted using GraphPad Prism software (version 8.0) to evaluate differences between groups. Spearman analyses were performed to assess the correlation between the relative expression levels of biomarkers and key iron parameters. A P-value < 0.05 was considered statistically significant.

## RESULTS

### Dimension reduction and single-cell type annotation

**Fig. 1:**
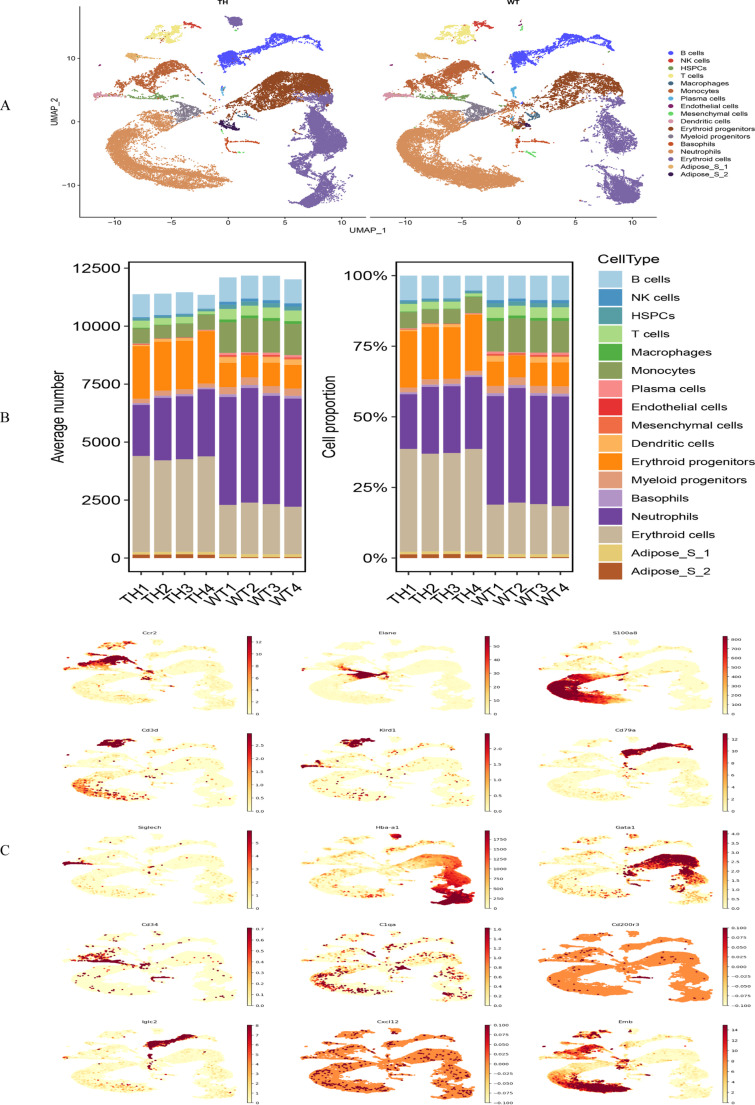
Dimension reduction and single-cell type annotation. **A**: Results of cell clustering annotation based on the Uniform Manifold Approximation and Projection (UMPA) algorithm; **B**: The proportional changes of different cell populations; **C**: The expression patterns of major cell marker genes demonstrated based on the UMPA algorithm

The UMAP algorithm was used for dimensionality reduction visualization, resulting in 47 initial cell subsets (Figure S3A). The validity of the clustering was verified using a heatmap of highly variable genes (HVGs) expression profiles (Figure S3B). Cell type annotation was performed using the SingleR package for automated classification (Figure S3C), followed by manual refinement based on the following criteria (Figure S3D): (1) marker genes were required to meet both conditions—average expression level > 1 log₂ counts per million and expression detected in > 30% of the cells; (2) for ambiguously annotated Subsets 27 and 30, gene ontology enrichment analysis was conducted using the clusterProfiler package (v4.0.5), after filtering out tissue-specific gene interference; (3) based on gene ontology enrichment results (adjusted *P* < 0.05) and sample tissue characteristics, these subsets were tentatively identified as adipose stromal cell subsets, named Adipose_S1 (enriched in lipid metabolism-related genes) and Adipose_S2 (enriched in extracellular matrix remodeling genes), respectively. After rigorous annotation and filtering, 17 biologically relevant cell subsets were identified (Fig. 1A). Quantitative analysis revealed a decrease in immune cells, including NK cells, T cells, macrophages, neutrophils, and monocytes, in the TDT group, while the proportion of erythroid cells increased (Fig. 1B). The expression patterns of representative marker genes are displayed in Fig. 1C.

### RNA velocity and cell communication

**Fig. 2:**
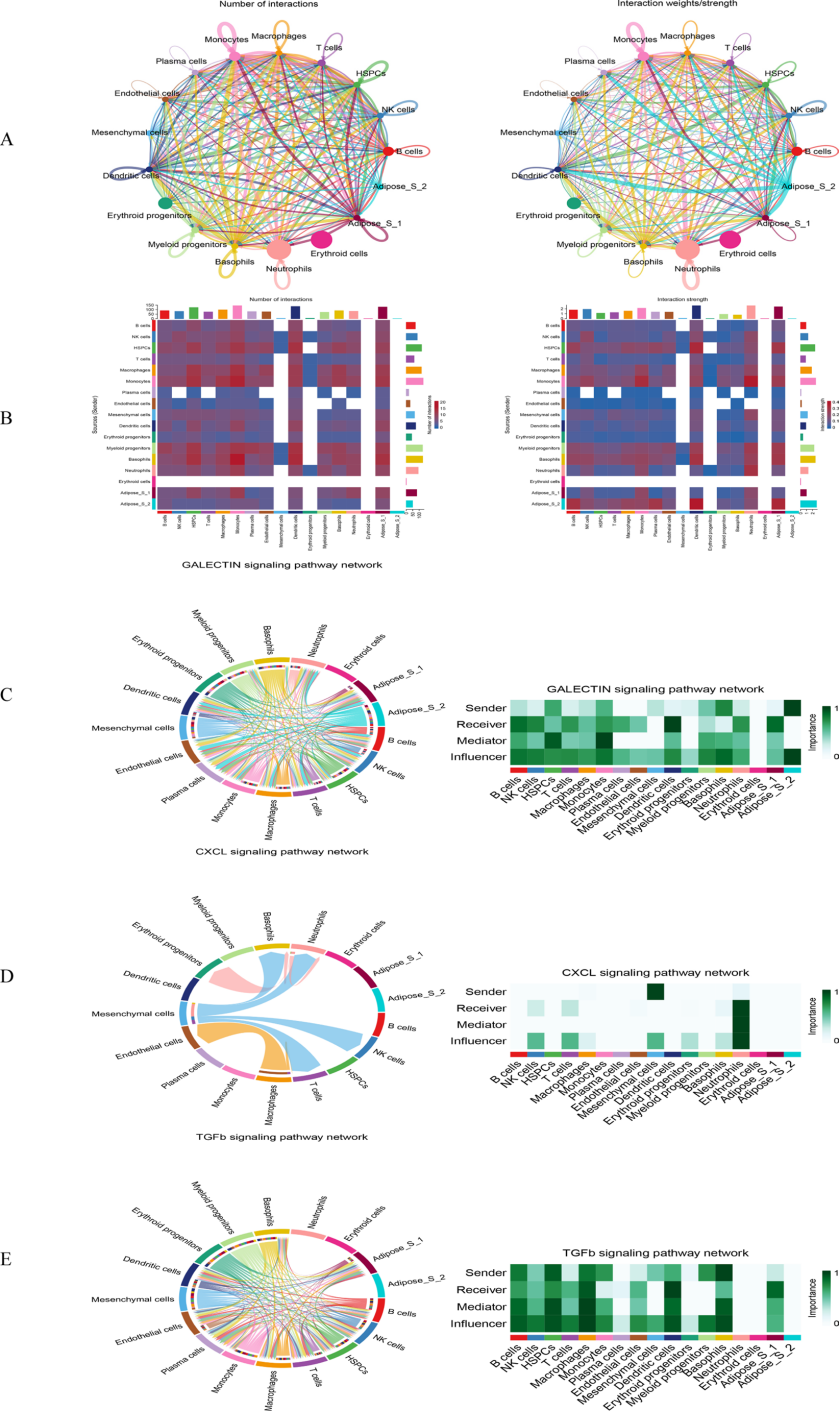
Cell communication. **A**: The number (left) and intensity (right) of communications between cell subpopulations; **B**: Heat maps displaying the number (left) and intensity (right) of communications between cell subpopulations. The color of the squares gradients from red to blue. In the left-hand graph, the number of interactions decreases from red to blue, while in the right-hand graph, it represents the intensity of interactions; **C**: The GALECTIN signaling pathway associated with the Erythroid cell subpopulation; **D**: The CXCL signaling pathway associated with the Erythroid cell subpopulation; **E**: The TGFb signaling pathway associated with the Erythroid cell subpopulation

The rate (velocity) vectors shown in Figure S4A were used for in-depth analysis of the cellular developmental trajectories. Figure S4B and S4C illustrate cell differentiation patterns in the TDT and control groups, respectively. To further explore intercellular interactions, we constructed an aggregated cell–cell communication network by quantifying the number of interactions or aggregating communication probabilities (Fig. 2A). Due to the complexity of these communication networks, we visualized the frequency and intensity of intercellular signals using heatmaps (Fig. 2B). In addition, we employed CellChat to analyze signaling pathway-specific communication, identifying major pathways such as GALECTIN, C-X-C motif chemokine ligand (CXCL), and transforming growth factor-beta (TGF-β) signaling (Fig. 2C-E).

### Cell subset analysis

**Fig. 3:**
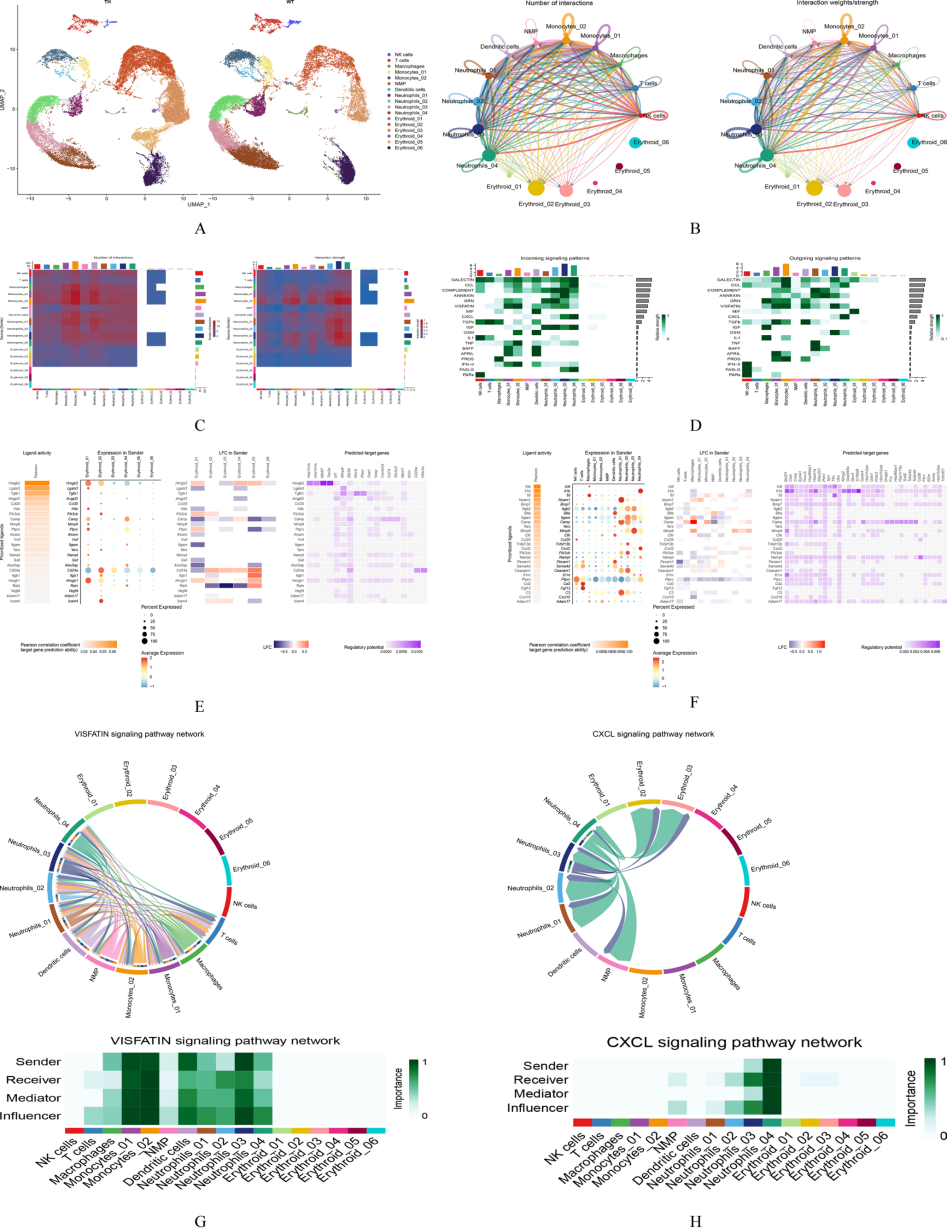
Cell communication analysis in cell subset. **A**: Re-clustering and annotation of the Erythroid-immune cell subpopulations; **B**: The number (left) and intensity (right) of communications between cell subpopulations; **C**: Heat maps showing the number (left) and intensity (right) of communications between cell subpopulations. The color of the squares gradually changes from red to blue. In the left graph, the number of interactions decreases from red to blue, and in the right graph, the intensity of interactions decreases from red to blue; **D**: The relative contributions of each cell type to the signaling pathway under specific signaling pathways in the signal-sending (left) and signal-receiving (right) modes; **E**: The ligands’ activity ranking of the signals sent by the Erythroid cell subpopulation, and prediction of its possible receptors; **F**: The ligands’ activity ranking of the signals sent by the immune cell subpopulation, and prediction of its possible receptors; **G**: The interactions between the immune cell subpopulation and the Erythroid cell subpopulation in the VISFATIN signaling pathway; H: The interactions between the immune cell subpopulation and the Erythroid cell subpopulation in the CXCL signaling pathway

Cells that exhibited the strongest communication with erythroid cells were subsets of NK cells, T cells, macrophages, monocytes, and neutrophils. To further investigate the mechanisms underlying disease onset and progression, these immune cell subsets and erythroid cells were extracted using the Seurat package for in-depth single-cell analysis. Cell clustering and annotation were performed, as shown in Fig. 3A. Intercellular communication between immune cells and erythroid subsets was analyzed by evaluating ligand–receptor interactions and communication states. Figure 3B and C display the communication frequency and intensity among the cell subsets. Figure 3D illustrates the relative contribution of each cell subset to specific signaling pathways in both signal-sending (left) and signal-receiving (right) modes. To identify active signaling molecules, the NicheNet package was used to rank active ligands when immune and erythroid cells acted as signal senders (Fig. 3E and F). These results were integrated with ligand–receptor predictions obtained from the CellChat package to identify signaling pathways specifically related to erythroid cells. Notably, among all erythroid subsets, only *Erythroid_02* and *Erythroid_03* demonstrated significant communication with the neutrophil subset through the CXCL signaling pathway. In addition, a distinct subset, *Erythroid_04*, identified specifically in the TH group, along with its downstream subset, *Erythroid_05*, and the previously mentioned *Erythroid_02* and *Erythroid_03*, were selected as four key erythroid cell nodes for subsequent analysis (Fig. 3G and H).

**Fig. 4:**
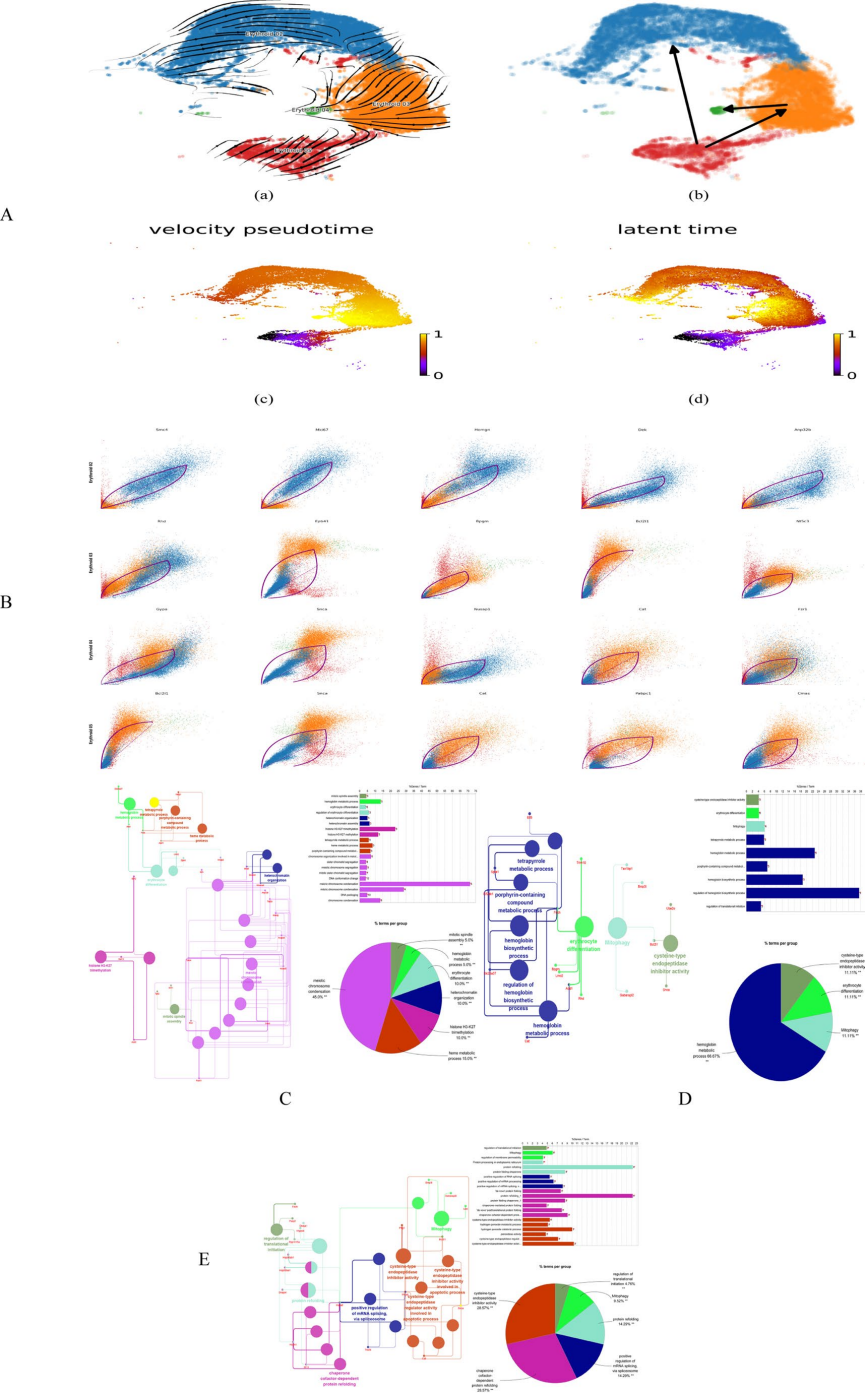
Key cell subsets analyses. **A**: Prediction of the cellular RNA velocity, differentiation time, and differentiation direction for the *Erythroid_02–05* subpopulation; **B**: The top 5 driver genes of the *Erythroid_02–05* subpopulation; C: Pathway enrichment analysis of the driver genes in the *Erythroid_02* cell subpopulation; D: Pathway enrichment analysis of the driver genes in the *Erythroid_03* cell subpopulation; E: Pathway enrichment analysis of the driver genes in the *Erythroid_05* cell subpopulation

We extracted four key erythroid cell subsets located at differentiation branch points and analyzed RNA velocity, latent time, and pseudotime trajectories (Fig. 4A). Using the scVelo framework, we then calculated gene- and cell-specific probabilities under the model’s optimal latent time and transcriptional state conditions. This analysis aimed to assess how accurately the learned spliced/unspliced RNA dynamics described the developmental trajectories of the cells (Fig. 4B). To identify genes driving erythroid differentiation, we ranked driver genes inferred by scVelo based on their splicing kinetics and goodness-of-fit. The top 50 genes from *Erythroid_02*,* Erythroid_03*, and *Erythroid_05* were subsequently selected for functional enrichment analysis.

The pathways enriched by the driver genes identified in the *Erythroid_02* cell subset included mitotic spindle assembly, hemoglobin metabolic processes, erythrocyte differentiation, regulation of erythrocyte differentiation, heterochromatin organization, heterochromatin assembly, histone H3-K27 trimethylation, histone H3-K27 methylation, tetrapyrrole metabolic process, heme metabolic process, porphyrin-containing compound metabolic process, chromosome organization involved in the meiotic cell cycle, and sister chromatid separation.

Among these, the pathways involving hemoglobin, tetrapyrrole, heme, and porphyrin-containing compound metabolism are particularly relevant to iron metabolism. The key genes associated with these pathways were *Atpif1*, *Fech*, *Hepb1*, *Add1*, *Hmgb2*, *Lmo2*, and *Zfpm1* (Fig. 4C).

The pathways enriched by the driver genes identified in the *Erythroid_03* cell subset included cysteine-type endopeptidase inhibitor activity, erythrocyte differentiation, mitophagy, tetrapyrrole metabolic process, hemoglobin metabolic process, porphyrin-containing compound metabolic process, hemoglobin biosynthesis process, regulation of hemoglobin biosynthesis process, and regulation of translation initiation. Among these, the tetrapyrrole metabolic process, hemoglobin metabolic process, porphyrin-containing compound metabolic process, and hemoglobin biosynthetic process are closely associated with iron metabolism. The key genes involved in these pathways were *Eif2ak1*, *Fech*, *Spta1*, *Add1*, *Cat*, and *Slc25a37* (Fig. 4D).

The pathways enriched by the driver genes in the *Erythroid_05* cell subset included regulation of translation initiation, mitophagy, membrane permeability, protein processing in the endoplasmic reticulum, cysteine-type endopeptidase inhibitor activity, hydrogen peroxide metabolic process, hydrogen peroxide catabolic process, peroxidase activity, cysteine-type endopeptidase regulatory activity involved in the apoptotic process, and cysteine-type endopeptidase inhibitor activity involved in the apoptotic process.

Among these, cysteine-type endopeptidase regulatory activity involved in the apoptotic process is closely associated with ferroptosis. In the thiol-dependent redox system, endogenous cysteine is primarily generated through the reduction of cystine, a process mediated by glutathione-thioredoxin reductase 1, which in turn regulates ferroptosis [13, 14]. The genes associated with this pathway included *Bcl2l1*,* Prdx6*, and *Snca* (Fig. 4E).

### Experimental validation

#### Viability of primary hepatocytes and iron ion staining

**Fig. 5:**
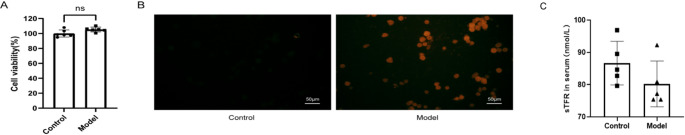
Viability of primary hepatocytes and iron ion staining. **A**: There was no significant difference in the viability of primary hepatocytes between thalassemia model mice and normal mice; **B**: The fluorescence signal of iron ion staining in primary hepatocytes of thalassemia model mice was stronger compared with that in normal mice; **C**: There was no significant difference in the serum soluble transferrin receptor (sTFR) level between thalassemia model mice and normal mice

Compared to normal mice, no significant difference was observed in the viability of primary hepatocytes from thalassemia model mice (Fig. 5A). The fluorescence signal of iron ion staining in primary hepatocytes from thalassemia model mice was significantly stronger than that in normal mice (Fig. 5B). Moreover, there was no significant difference in serum soluble transferrin receptor levels between thalassemia model mice and normal mice (Fig. 5C).

#### Oxidative stress level in liver tissue

**Fig. 6:**
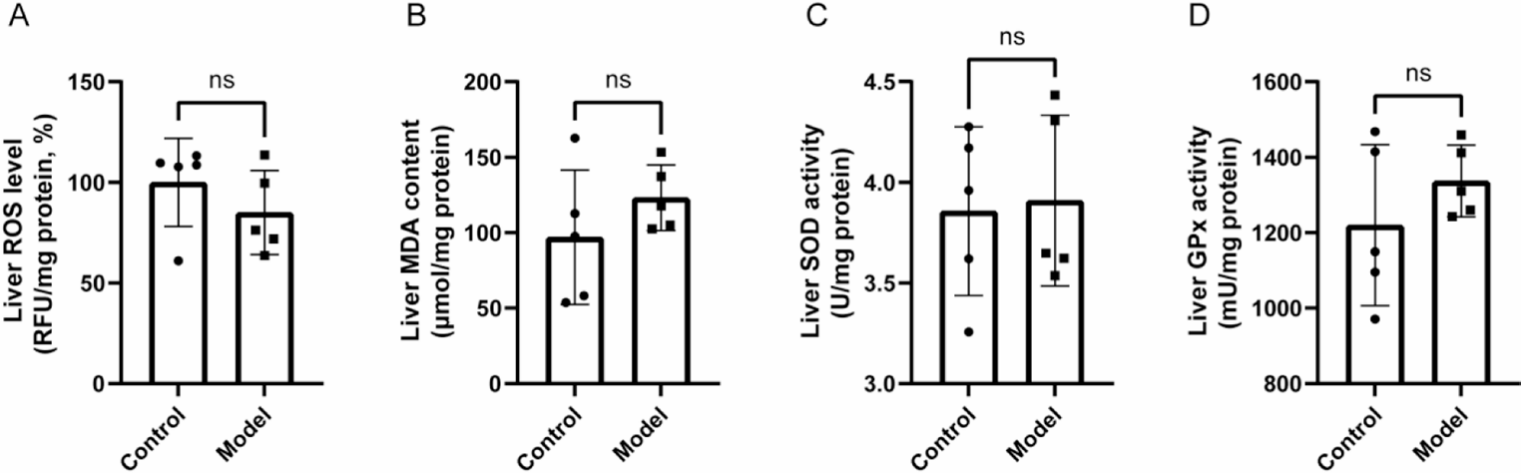
Comparison of related oxidative stress indices in the liver tissues between thalassemia model mice and normal mice. **A**: Reactive oxygen species (ROS) level; **B**: Malondialdehyde (MDA) content; **C**: Superoxide dismutase (SOD) activity; **D**: Glutathione peroxidase (GPx) activity

Compared with normal mice, there were no significant differences in ROS levels, malondialdehyde content, superoxide dismutase activity, and glutathione peroxidase activity in the liver tissue of thalassemia model mice (Fig. 6A-D).

### SI metabolism indices and iron content in liver tissue

**Fig. 7:**
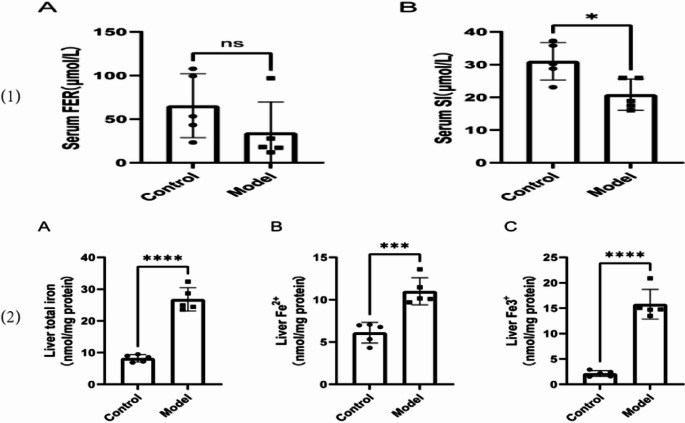
Serum iron metabolism indices and iron content in liver tissue. (**1**): The serum FER and SI levels; (**2**): The contents of total iron, Fe^2+^ and Fe^3+^ in the liver tissue

Compared to normal mice, there was no significant difference in serum ferritin levels in thalassemia model mice. In contrast, serum SI levels in thalassemia model mice were significantly decreased compared to normal mice (Fig. 7 [1]). Furthermore, the contents of total iron, Fe²⁺, and Fe³⁺ in liver tissue were significantly increased in thalassemia model mice relative to normal mice (Fig. 7 [2]).

#### RT-PCR

**Fig. 8:**
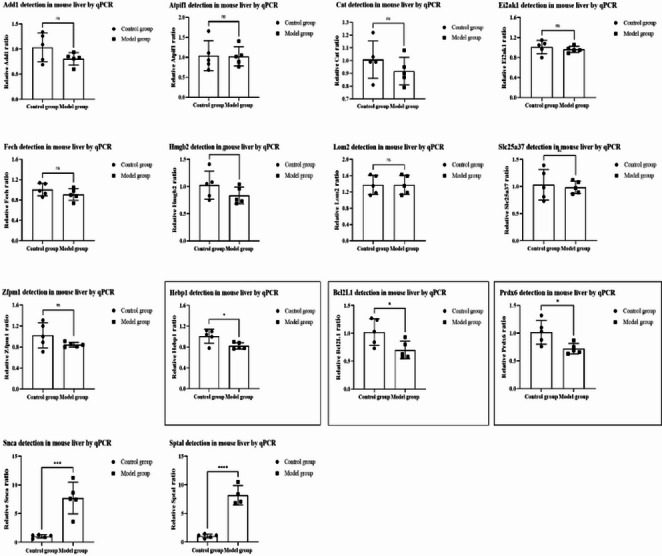
The expression of *Add1*,* Atpif1*,* Cat*,* Eif2ak1*,* Fech*,* Hmgb2*,* Lmo2*,* Slc25a37*,* Zfpm1*,* Hepb1*,* BCL2L1*,* Prdx6*,* Snca*, and *Spta1*

There were no significant differences between the thalassemia and control models in the expression of *Add1*, *Atpif1*,* Cat*,* Eif2ak1*,* Fech*,* Hmgb2*,* Lmo2*,* Slc25a37*, and *Zfpm1*. However, in the thalassemia model group, the expression of *BCL2L1*, *Hepb1*, and *Prdx6* was significantly downregulated, whereas the expression of *Spta1* and *Snca* was significantly upregulated (Fig. 8). The differential expression of these genes (*BCL2L1*, *Hepb1*, *Prdx6* downregulation; *Spta1*, *Snca* upregulation) in TDT model mice was significantly associated with elevated hepatic iron levels (total iron, Fe²⁺, Fe³⁺) and reduced SI, supporting their potential as functional biomarkers of iron overload complications.

#### Correlation between biomarker expression and iron parameters

To investigate the relationship between the identified biomarkers and iron metabolism dysfunction, we performed Spearman correlation analyses between the relative expression levels of BCL2L1, Hepb1, Prdx6, Spta1, Snca and key iron parameters in TDT model mice and controls. BCL2L1 expression negatively correlated with hepatic total iron (*r* = −0.78, *p* = 0.002), Fe²⁺ (*r* = −0.75, *p* = 0.003), and Fe³⁺ (*r* = −0.72, *p* = 0.005), and positively correlated with SI (*r* = 0.69, *p* = 0.008); Hepb1 expression negatively correlated with hepatic total iron (*r* = −0.81, *p* = 0.001), Fe²⁺ (*r* = −0.79, *p* = 0.002), and Fe³⁺ (*r* = −0.76, *p* = 0.003), and positively correlated with SI (*r* = 0.73, *p* = 0.004); Prdx6 expression negatively correlated with hepatic total iron (*r* = −0.74, *p* = 0.004), Fe²⁺ (*r* = −0.71, *p* = 0.006), and Fe³⁺ (*r* = −0.68, *p* = 0.009), and positively correlated with SI (*r* = 0.65, *p* = 0.012); Spta1 expression positively correlated with hepatic total iron (*r* = 0.83, *p* < 0.001), Fe²⁺ (*r* = 0.80, *p* = 0.001), and Fe³⁺ (*r* = 0.77, *p* = 0.002), and negatively correlated with SI (*r* = −0.75, *p* = 0.003); Snca expression positively correlated with hepatic total iron (*r* = 0.76, *p* = 0.003), Fe²⁺ (*r* = 0.73, *p* = 0.004), and Fe³⁺ (*r* = 0.70, *p* = 0.007), and negatively correlated with SI (*r* = −0.67, *p* = 0.010). These results confirm strong associations between the identified biomarkers and iron overload phenotypes in TDT model mice.

## Discussion

In this study, we employed single-cell sequencing, bioinformatics analysis, and animal experiments to explore the cellular and molecular mechanisms related to TDT, yielding a series of valuable results. These findings provide novel insights into the pathogenesis of TDT. At the single-cell level, we characterized the composition and features of cell subsets through dimensionality reduction and annotation. In the TDT group, the proportion of immune cells was decreased, whereas the proportion of erythroid cells was increased. RNA velocity and cell communication analyses revealed that immune cell subsets, such as NK cells and T cells, communicate closely with erythroid cells. These interactions may play important roles in TDT pathogenesis. Further cell subset analysis focused on key cell nodes. The significant communication between *Erythroid_02*, *Erythroid_03*, and neutrophils via the CXCL signaling pathway, as well as between *Erythroid_04* and *Erythroid_05*, highlights critical nodes that provide specific entry points for studying the interaction between erythroid cells and immune cells. In terms of animal experiment verification, although there was no significant difference in the viability of primary hepatocytes from thalassemia model mice, the fluorescence signal of iron ion staining was markedly stronger, and the levels of total iron, Fe^2+,^ and Fe^3+^ in liver tissue were significantly elevated. These further underscore the close relationship between TDT and iron metabolism disorders. Subsequent validation of the 14 driver genes identified through bioinformatics revealed that the expression levels of *BCL2L1*,* Hepb1*, and *Prdx6* were significantly downregulated, whereas those of *Spta1* and *Snca* were significantly upregulated. These gene expression changes may contribute to the pathological mechanisms underlying TDT. Further investigation into these genes will facilitate a deeper understanding of TDT’s molecular basis.

We found that the proportion of immune cells significantly decreased, whereas that of erythroid cells increased in the TDT group. In patients undergoing TDT, erythrocytes exhibit abnormal metabolic processes. Hemoglobin degradation products released by these abnormal erythrocytes may activate the apoptotic signaling pathway within immune cells, leading to enhanced immune cell apoptosis and a consequent reduction in their proportion [15, 16]. Moreover, a chronic inflammatory state is present. Several inflammatory factors produced in this inflammatory microenvironment can impair immune cell survival and function, inducing apoptosis. Simultaneously, inflammation may also disrupt the proliferation and differentiation of immune cells, causing the rate of immune cell generation to fail to compensate for apoptosis, resulting in their decreased proportion [17, 18].

The increase in erythroid cells may be due to the persistent anemic state in patients with TDT, which activates the body’s hypoxia-sensing mechanism. In response, the kidneys secrete elevated levels of erythropoietin, which specifically targets erythroid progenitor cells to promote their proliferation, differentiation, and maturation, leading to massive erythroid cell production. Additionally, erythropoietin may indirectly inhibit immune cell generation and development, contributing to the relatively lower proportion of cells [19]. Lastly, TDT induces tissue hypoxia, activating the hypoxia-inducible factor pathway. Hypoxia-inducible factor upregulates many erythropoiesis-related genes, further enhancing erythroid cell proliferation and differentiation [20, 21].

Further analysis revealed immune cell subsets that communicate closely with erythrocytes, such as NK cells and T cells. Through the pathway-specific analyses, we identified the GALECTIN, CXCL, and TGF-β signaling pathways as key mediators involved in these interactions, highlighting their potential as therapeutic targets. During erythrocyte-immune cell interactions, GALECTIN proteins mediate cell-to-cell adhesion by recognizing specific glycosylated ligands on the surfaces of erythrocytes, NK cells, and T cells. This adhesion facilitates close cellular contact, establishing a foundation for subsequent signal transduction and functional regulation [22]. CXCL chemokines regulate extracellular matrix components and the cytokine network within hematopoietic tissues, such as bone marrow, thereby influencing the proliferation, differentiation, and maturation of erythroid cells. Concurrently, CXCL signaling affects the distribution and function of immune cells within the hematopoietic microenvironment, indirectly modulating erythrocyte-immune cell interactions [23]. In pathological conditions, abnormal erythrocytes may induce tissue damage and inflammatory responses. The TGF-β signaling pathway contributes to creating a favorable microenvironment for the survival and function of both erythrocytes and immune cells by regulating extracellular matrix deposition and remodeling. Moreover, it influences interactions and signal transduction processes [24].

The iron metabolism-related pathways and genes in *Erythroid _02* and *Erythroid _03* suggested that abnormal iron metabolism may play a crucial role in TDT. The pathways related to ferroptosis in *Erythroid_05* indicated the potential role of cell death in the pathogenesis. In animal experiments, the fluorescence signal of iron ion staining in the thalassemia model mice was significantly stronger, and the contents of total iron, Fe^2+,^ and Fe^3+^ in the liver tissue were significantly increased. This further confirms the close relationship between TDT and iron metabolism disorders. Among the SI metabolism indicators, the significant decrease in the SI level also reflects an imbalance in iron homeostasis in patients with TDT. During thalassemia, owing to impaired globin synthesis, ineffective erythropoiesis increases. Several immature red blood cells undergo apoptosis in the bone marrow, and these apoptotic cells release large amounts of iron, resulting in impaired utilization [25]. At the same time, to compensate for the hypoxic state caused by anemia, the body increases the absorption of iron in the intestine. However, the absorbed iron cannot be effectively used to synthesize hemoglobin, leading to a large amount of iron deposition in tissues and organs, such as the liver, manifested as an increase in the contents of total iron, Fe^2+^ and Fe^3+^ in the liver tissue and an enhanced fluorescence signal of iron-ion staining [26]. The significant decrease in the SI level occurs because a large amount of iron is “trapped” in tissues and cannot enter the blood circulation normally, which reflects the imbalance of iron homeostasis in patients with TDT and further confirms the close relationship between TDT and iron metabolism disorders [27].

Further animal experimental verification revealed that, among the 14 driver genes explored, the expression levels of *BCL2L1*,* Hepb1*, and *Prdx6* were significantly downregulated in the TDT model group, whereas the expression levels of *Spta1* and *Snca* were significantly upregulated. The strong correlations between biomarker expression and iron parameters further validate their role in iron metabolism dysregulation. Abnormal conditions, such as iron metabolism disorders and oxidative stress induced by TDT, can have a wide range of effects on intracellular signaling pathways and gene regulation [28]. Excessive iron deposition may induce oxidative damage, activate a series of stress signaling pathways, and inhibit the expression of anti-apoptotic genes, such as *BCL2L1*, increasing the tendency of cells to undergo apoptosis [29]. The negative correlation of *BCL2L1* with hepatic iron aligns with its anti-apoptotic function, as iron overload-induced oxidative stress may suppress *BCL2L1* to promote cell death in iron-loaded tissues. Regarding *Hebp1*, iron overload may interfere with the transcriptional regulation or mRNA stability, leading to the downregulation of its expression and affecting the regulation and storage of iron metabolism [30]. *Hepb1*, a key iron regulatory gene, showed the most robust negative correlation with hepatic iron accumulation, supporting its role in modulating intracellular iron trafficking—its downregulation likely exacerbates iron deposition in hepatocytes. Downregulation of *Prdx6* expression occurs because oxidative stress exceeds its antioxidant capacity, and the body’s feedback regulation reduces its expression [31]. *Prdx6* as an antioxidant enzyme, showed a negative correlation with hepatic iron, consistent with its downregulation being a consequence of overwhelmed antioxidant capacity in iron-overloaded conditions. The upregulation of *Spta1* is a compensatory response that maintains the stability of the red blood cell membrane in an abnormal cellular environment. The body attempts to strengthen the damaged red blood cell membrane structure by increasing *Spta1* [32]. The upregulation of *Snca* may be related to the stress response of the nervous system to the pathological state associated with TDT, and is involved in the adaptive regulation of nerve cells to damage, such as iron deposition and oxidative stress [33]. *Spta1* and Snca exhibited strong positive correlations with hepatic iron, reflecting compensatory responses: *Spta1* upregulation stabilizes erythrocyte membranes damaged by iron-induced oxidative stress, while Snca upregulation may mediate adaptive stress responses to iron deposition.

In addition, the reduced proportion of immune cells (NK cells, T cells, macrophages, neutrophils, monocytes) in TDT and their close communication with erythroid subsets (via GALECTIN, CXCL, and TGF-β pathways) are tightly linked to the differential expression of the identified biomarkers, as these genes regulate processes critical to immune cell survival, function, and interaction with erythroid cells. *BCL2L1* (a key anti-apoptotic gene) is downregulated in TDT mice. Our data show reduced immune cell proportions in TDT, which may partly result from enhanced apoptosis-a process regulated by *BCL2L1*. NK cells and T cells rely on *BCL2L1* to suppress mitochondrial apoptosis during activation and proliferation [34]. In TDT, iron overload-induced oxidative stress may downregulate *BCL2L1*, impairing immune cell survival and contributing to their reduced abundance. Additionally, *BCL2L1* dysfunction in erythroid cells (Erythroid_05) could alter ligand-receptor interactions with NK cells, further disrupting immune homeostasis [22]. *Hepb1*, a heme-binding protein involved in iron trafficking, is downregulated in TDT. Immune cells require iron for essential functions: macrophages use iron for erythrophagocytosis and heme recycling [35], while neutrophils depend on iron for oxidative burst-mediated pathogen clearance [36]. *Hepb1* downregulation may impair intracellular iron distribution, reducing macrophage efficiency in clearing damaged erythrocytes (a hallmark of TDT) and diminishing neutrophil antimicrobial capacity. This dysfunction could perpetuate chronic inflammation, as uncleared erythrocyte debris triggers pro-inflammatory signaling [37]. *Prdx6*, an antioxidant enzyme with peroxidase activity, is downregulated in TDT. Immune cells, particularly neutrophils and monocytes, generate ROS during phagocytosis, but they rely on antioxidants like *Prdx6* to prevent self-damage [38]. In TDT, iron overload exacerbates ROS production [4], and *Prdx6* downregulation likely impairs immune cells’ ability to mitigate oxidative stress. This may increase neutrophil/monocyte apoptosis and reduce their proportional abundance, as observed in our TDT model. Abnormal erythrocytes in TDT exhibit membrane fragility due to globin chain imbalance, and *Spta1* upregulation likely represents a compensatory attempt to maintain membrane integrity [32]. However, damaged erythrocytes still release heme and iron-rich debris, which act as damage-associated molecular patterns (DAMPs) [39]. These DAMPs activate macrophages and neutrophils via TLR4 signaling [40], potentially altering their functional state and contributing to their reduced proportions through exhaustion or apoptosis. *Snca*, upregulated in TDT, is increasingly recognized for its role in immune regulation. *Snca* can modulate macrophage polarization: in iron-overloaded conditions, *Snca* upregulation may shift macrophages toward a pro-inflammatory M1 phenotype [41], which could enhance erythrophagocytosis but also promote chronic inflammation. Additionally, *Snca* interacts with T cell receptors [42], and its upregulation might disrupt T cell activation, contributing to the reduced T cell proportions observed in TDT.

Our findings identify *BCL2L1*, *Hepb1*, *Prdx6*, *Spta1*, and *Snca* as potential biomarkers in TDT. Their differential expression correlates with key iron overload phenotypes in TDT mice, supporting their role as functional markers indicating iron metabolism dysregulation. Additionally, their transcriptional dysregulation distinguishes TDT from normal states, suggesting utility as diagnostic biomarkers. Together, these biomarkers link TDT pathogenesis to iron overload complications, offering targets for monitoring and potentially mitigating iron-related tissue damage.

## Supplementary information

Below is the link to the electronic supplementary material.

Supplementary figure 1 (PNG 489 KB)

High Resolution Image (TIF 18.4 MB)

Supplementary figure 2 (PNG 1.52 MB)

High Resolution Image (TIF 21.8 MB)

Supplementary figure 3 (PNG 1.73 MB)

High Resolution Image (TIF 24.1 MB)

Supplementary figure 4 (PNG 2.83 MB)

High Resolution Image (TIF 20.5 MB)

## Data Availability

No datasets were generated or analysed during the current study.
