# Supplementary figures and images for: Genetic biomarkers and crucial cell subsets of iron metabolism in Beta-Thalassemia: insights from bioinformatics and experimental validation
Source: Ann Hematol. 2025 Sep 16;104(9):4369–84. doi: 10.1007/s00277-025-06605-6 (PMC12552345; doi:10.1007/s00277-025-06605-6)

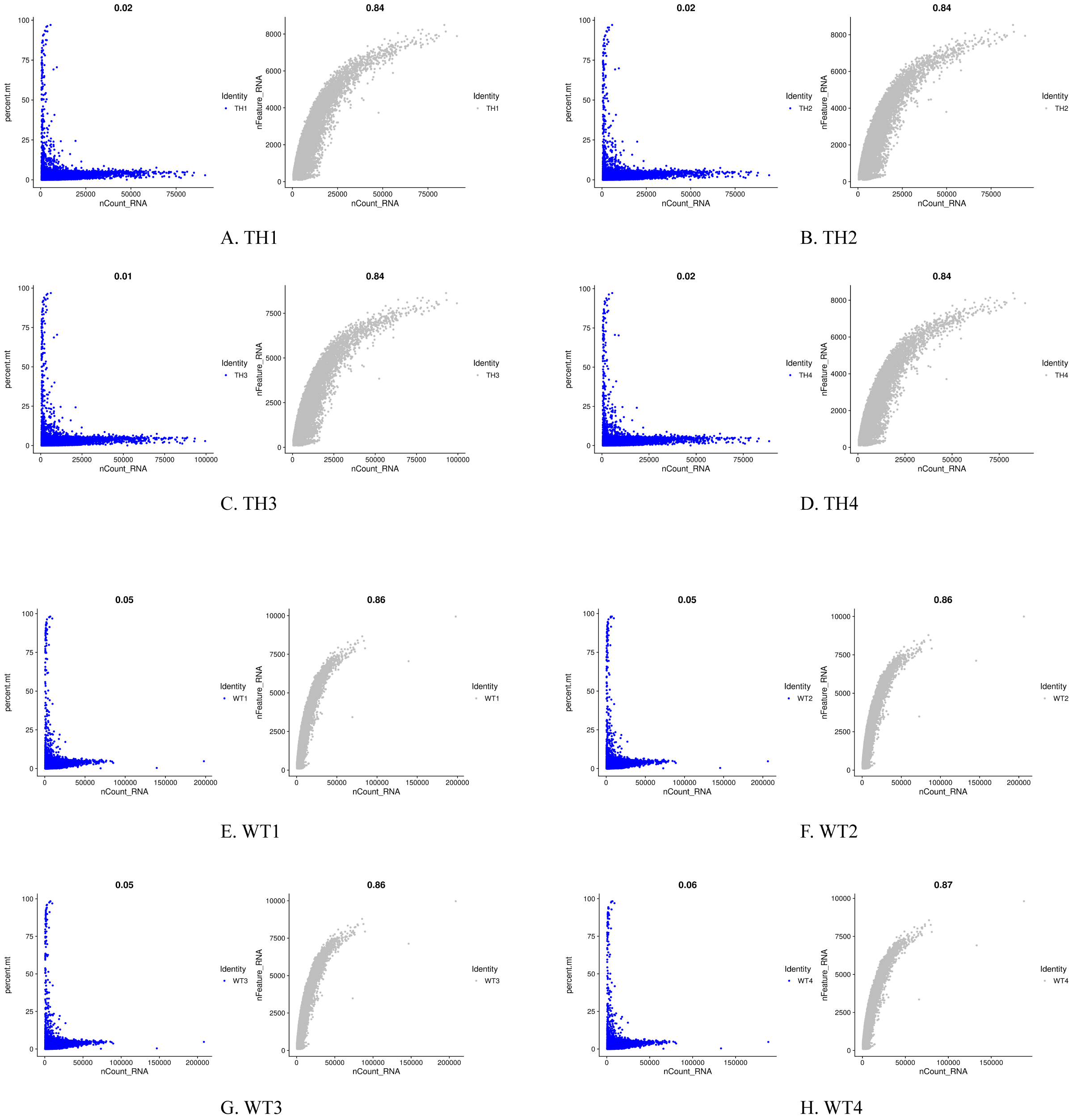

Supplement: Supplementary file 1 — (PNG 489 KB) [file 277_2025_6605_Fig9_ESM.png]

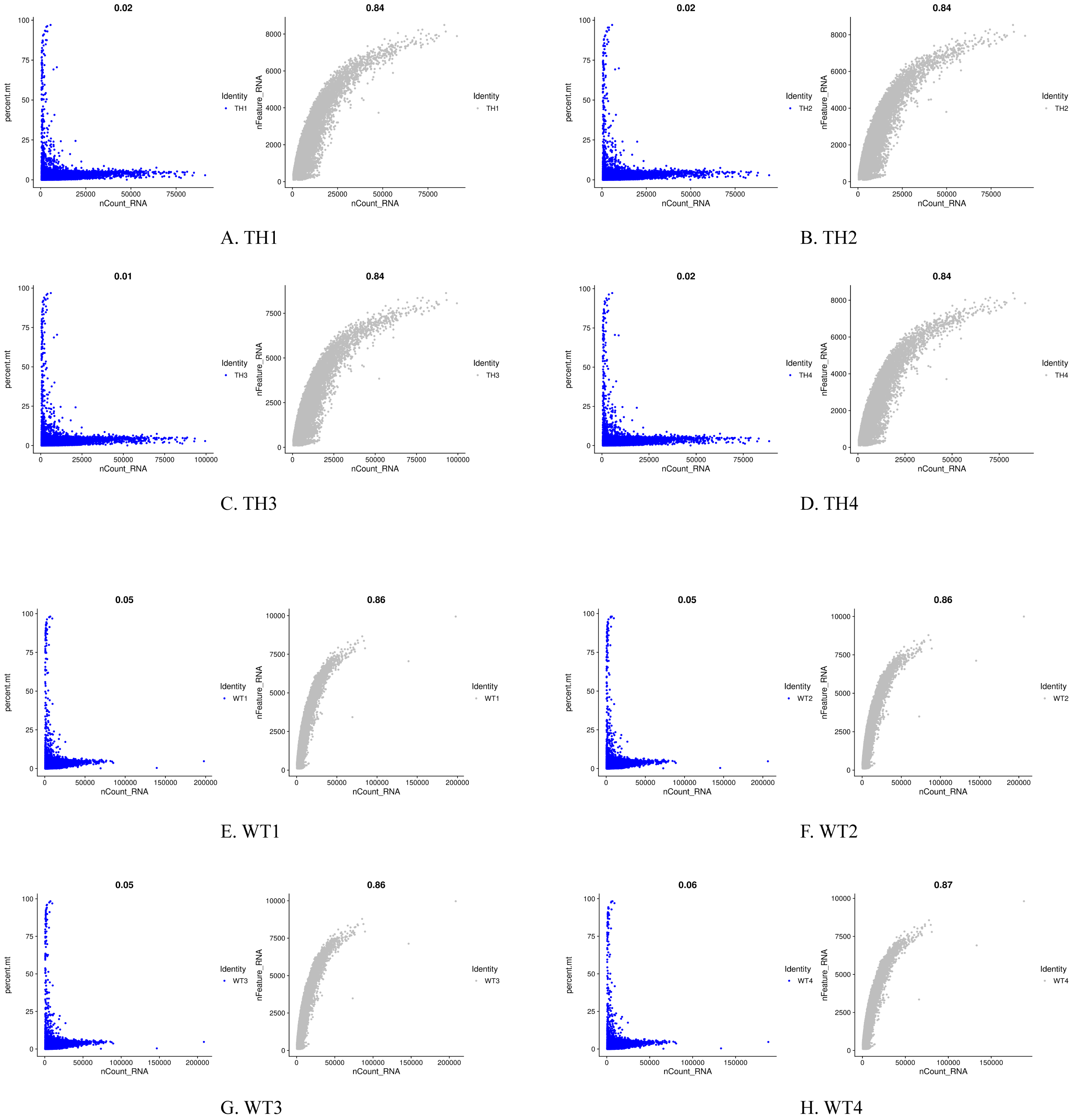

Supplement: Supplementary file 2 — High Resolution Image (TIF 18.4 MB) [file 277_2025_6605_MOESM1_ESM.tif]

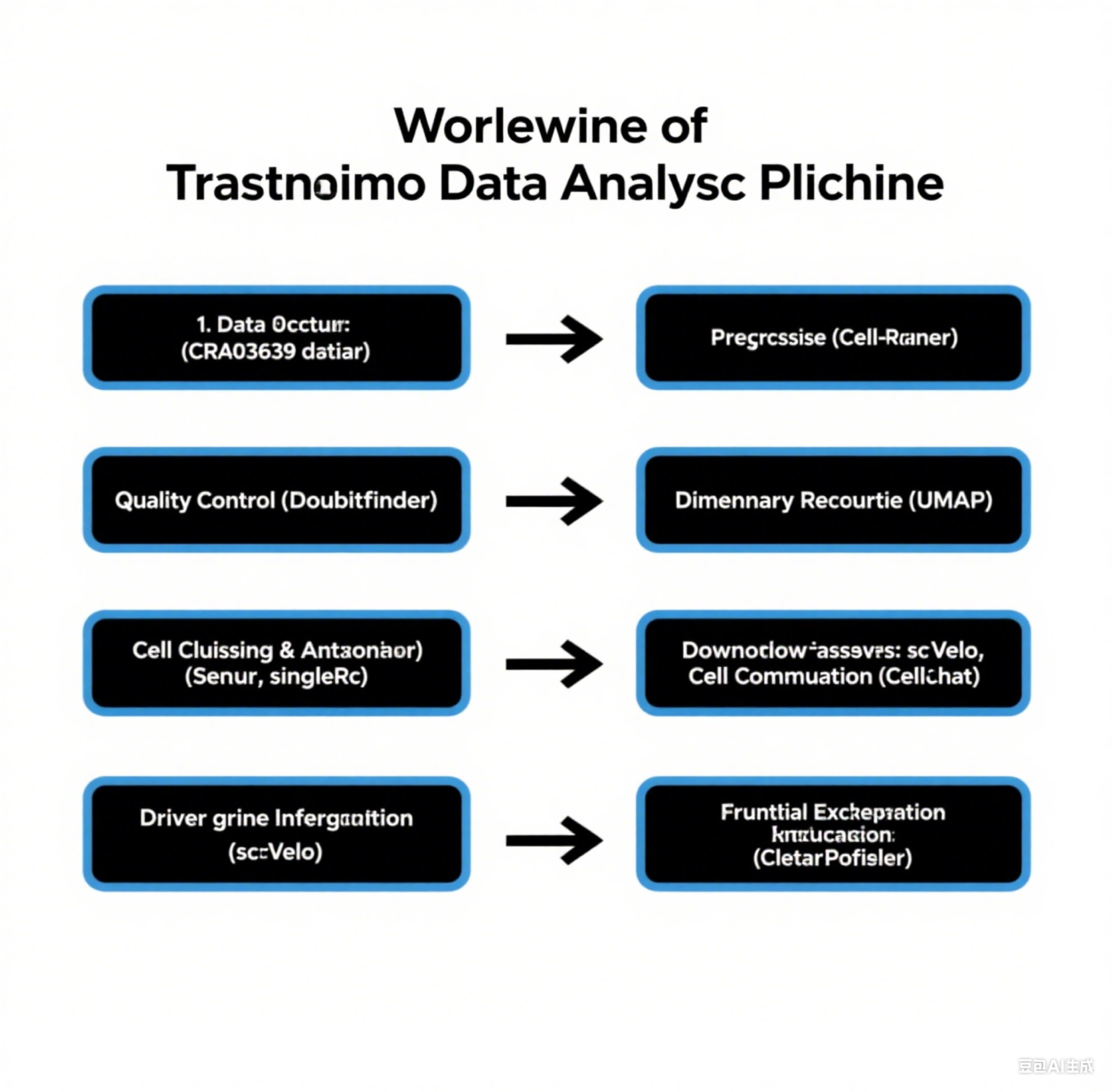

Supplement: Supplementary file 3 — (PNG 1.52 MB) [file 277_2025_6605_Fig10_ESM.png]

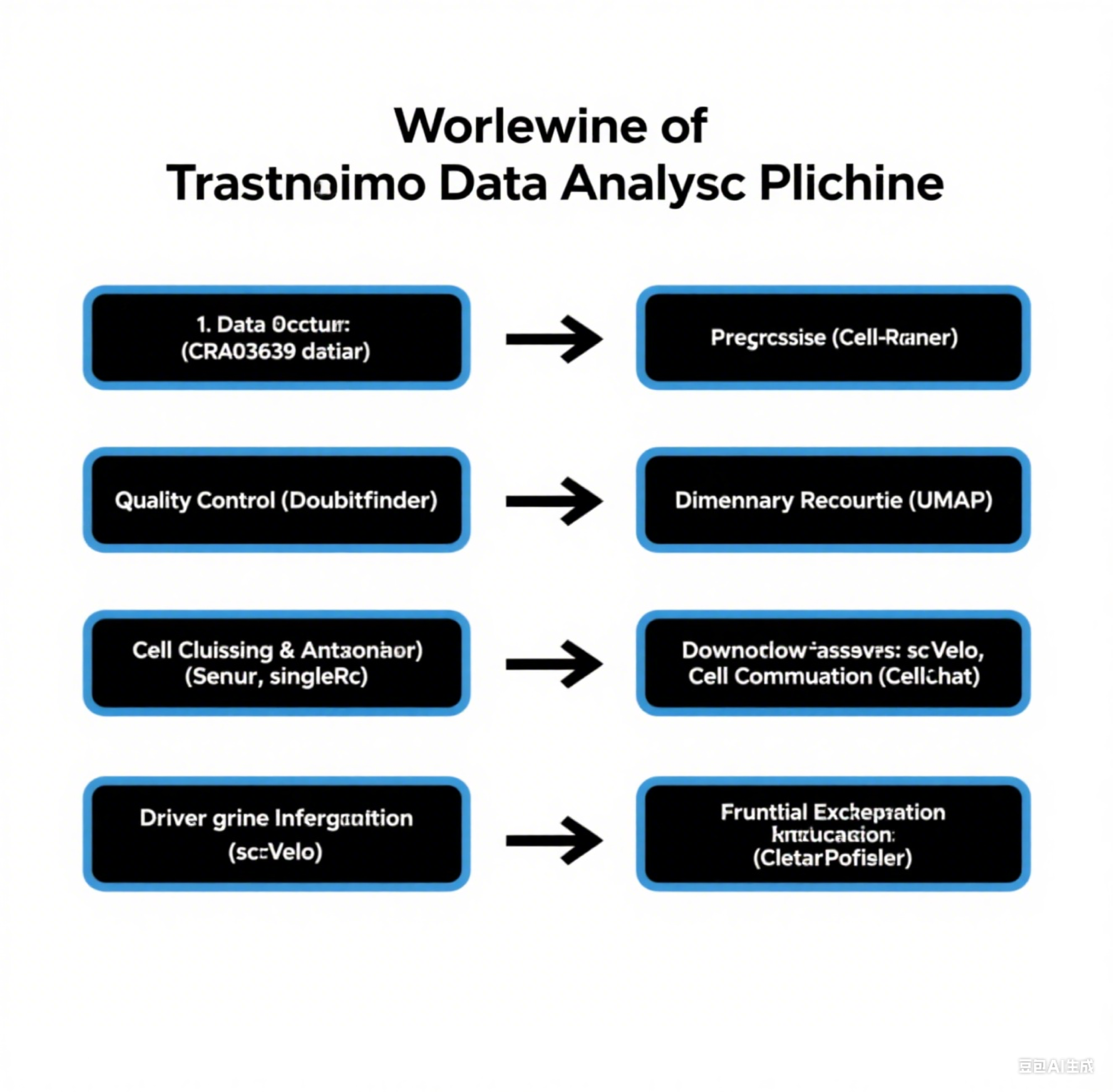

Supplement: Supplementary file 4 — High Resolution Image (TIF 21.8 MB) [file 277_2025_6605_MOESM2_ESM.tif]

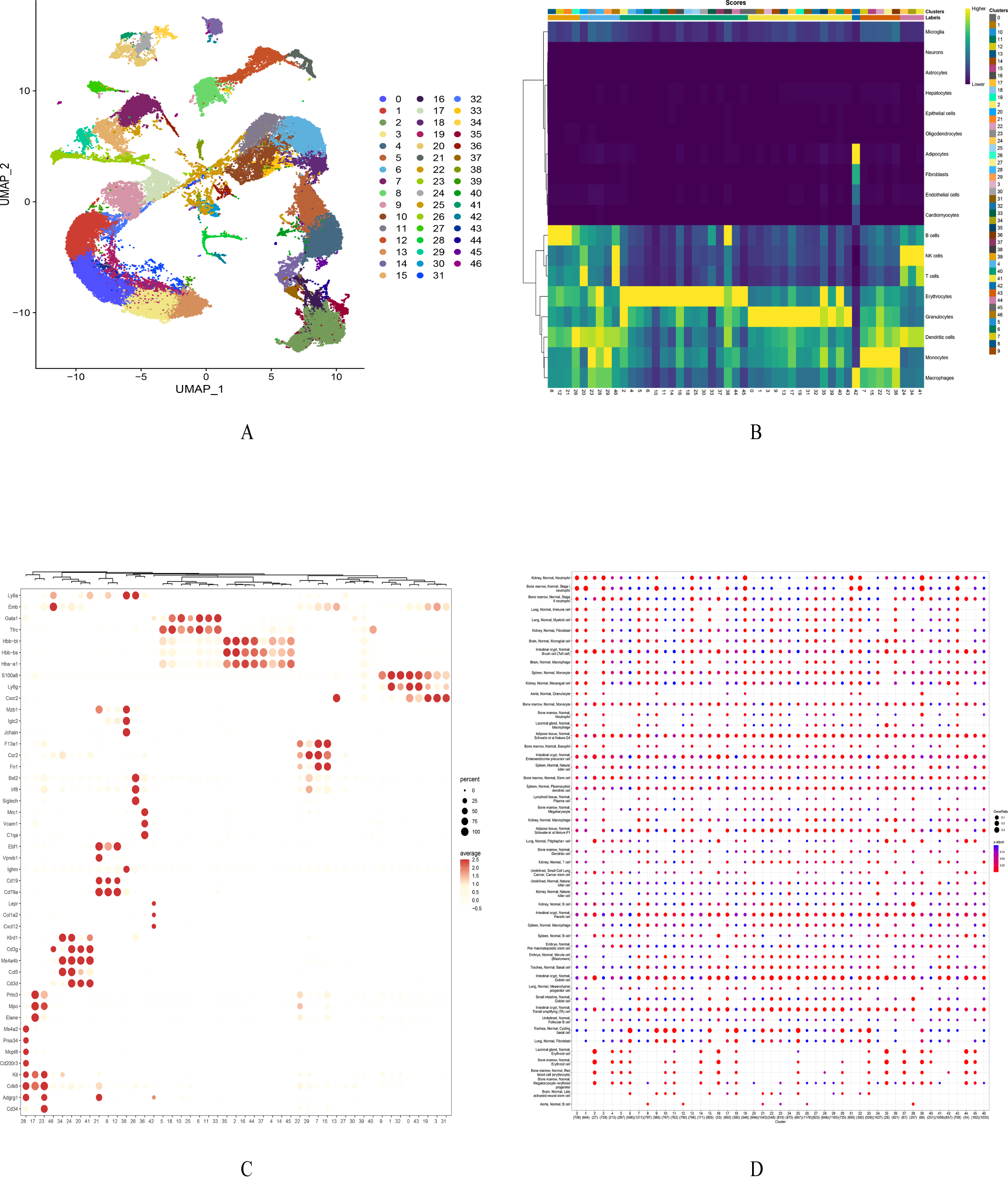

Supplement: Supplementary file 5 — (PNG 1.73 MB) [file 277_2025_6605_Fig11_ESM.png]

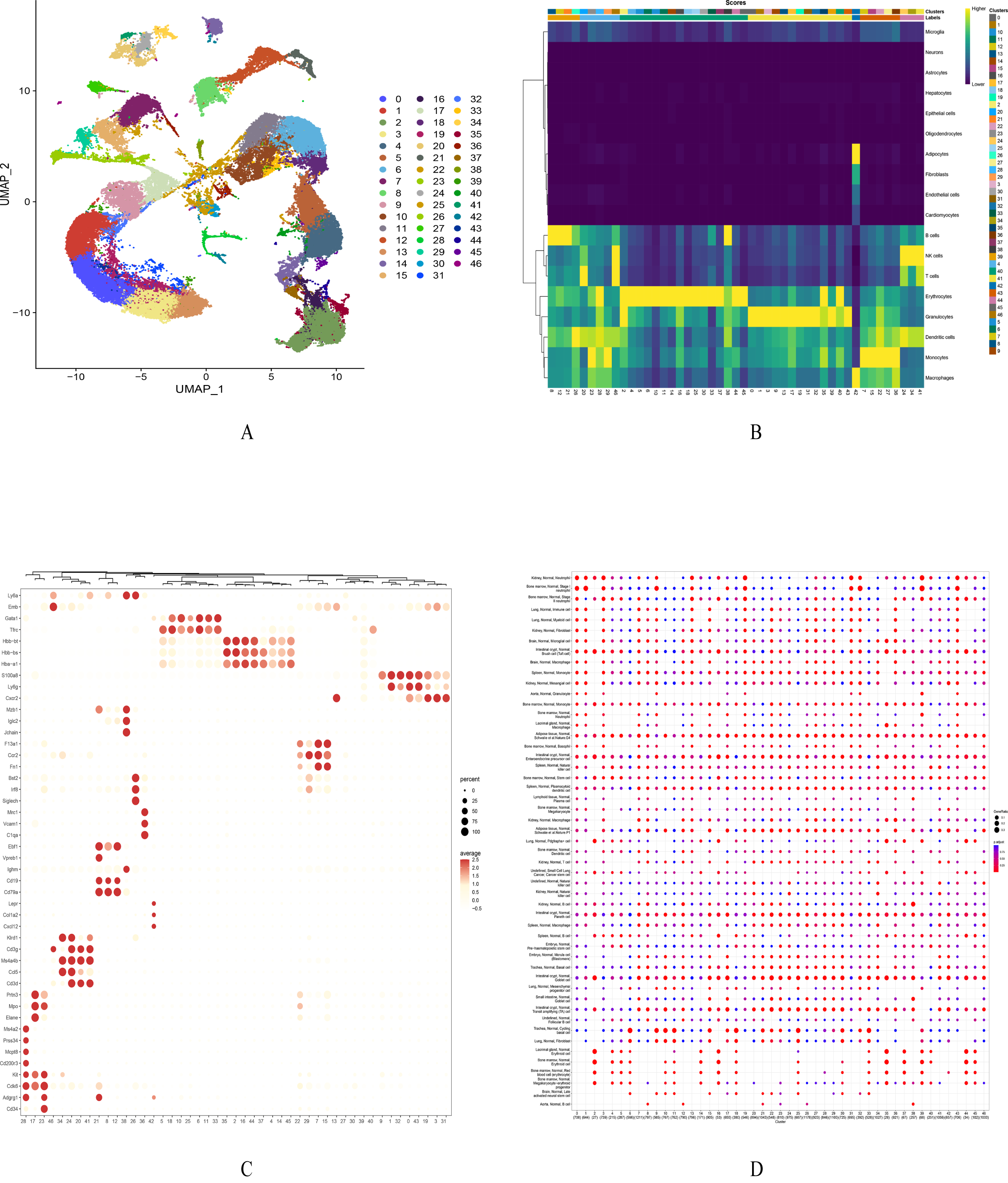

Supplement: Supplementary file 6 — High Resolution Image (TIF 24.1 MB) [file 277_2025_6605_MOESM3_ESM.tif]

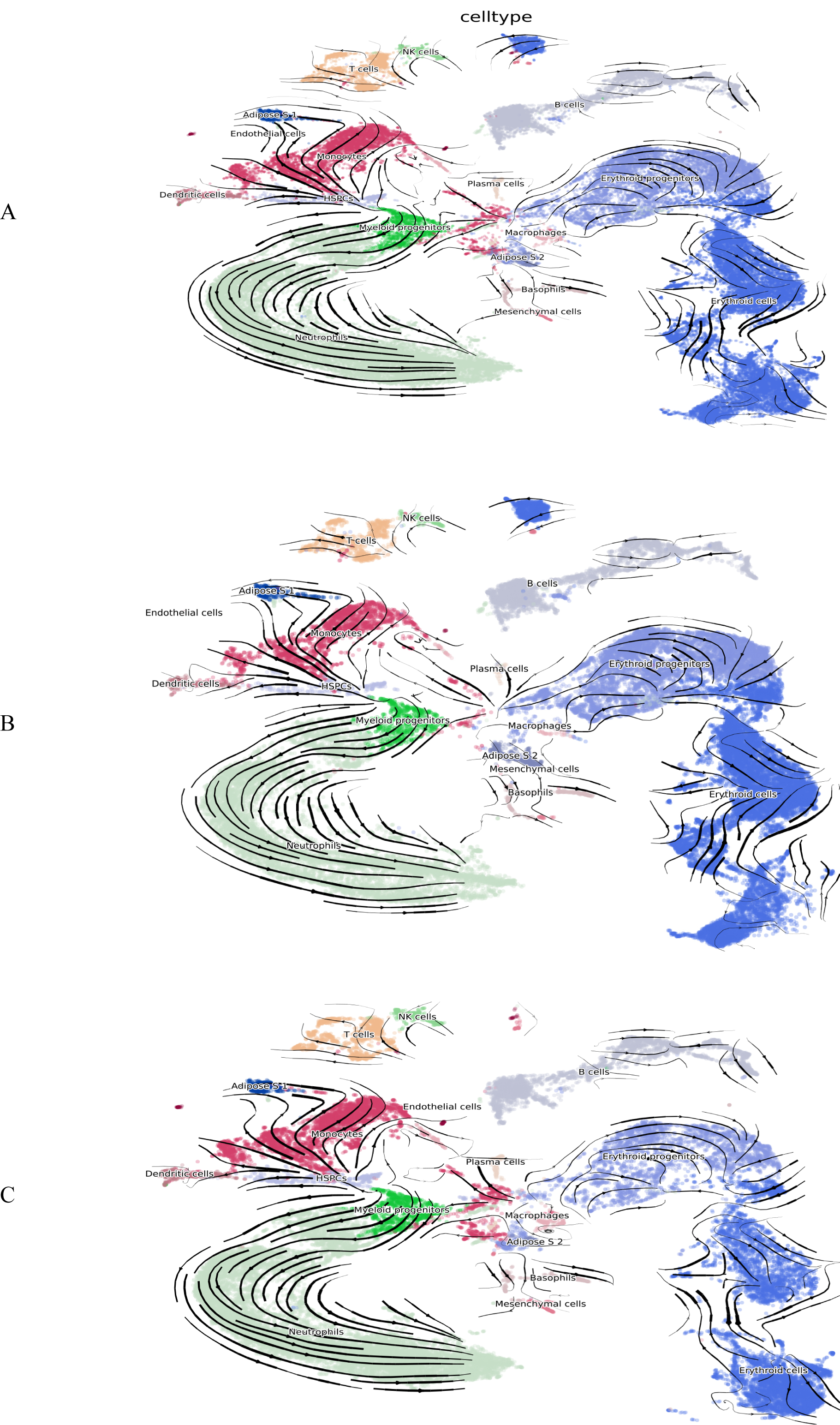

Supplement: Supplementary file 7 — (PNG 2.83 MB) [file 277_2025_6605_Fig12_ESM.png]

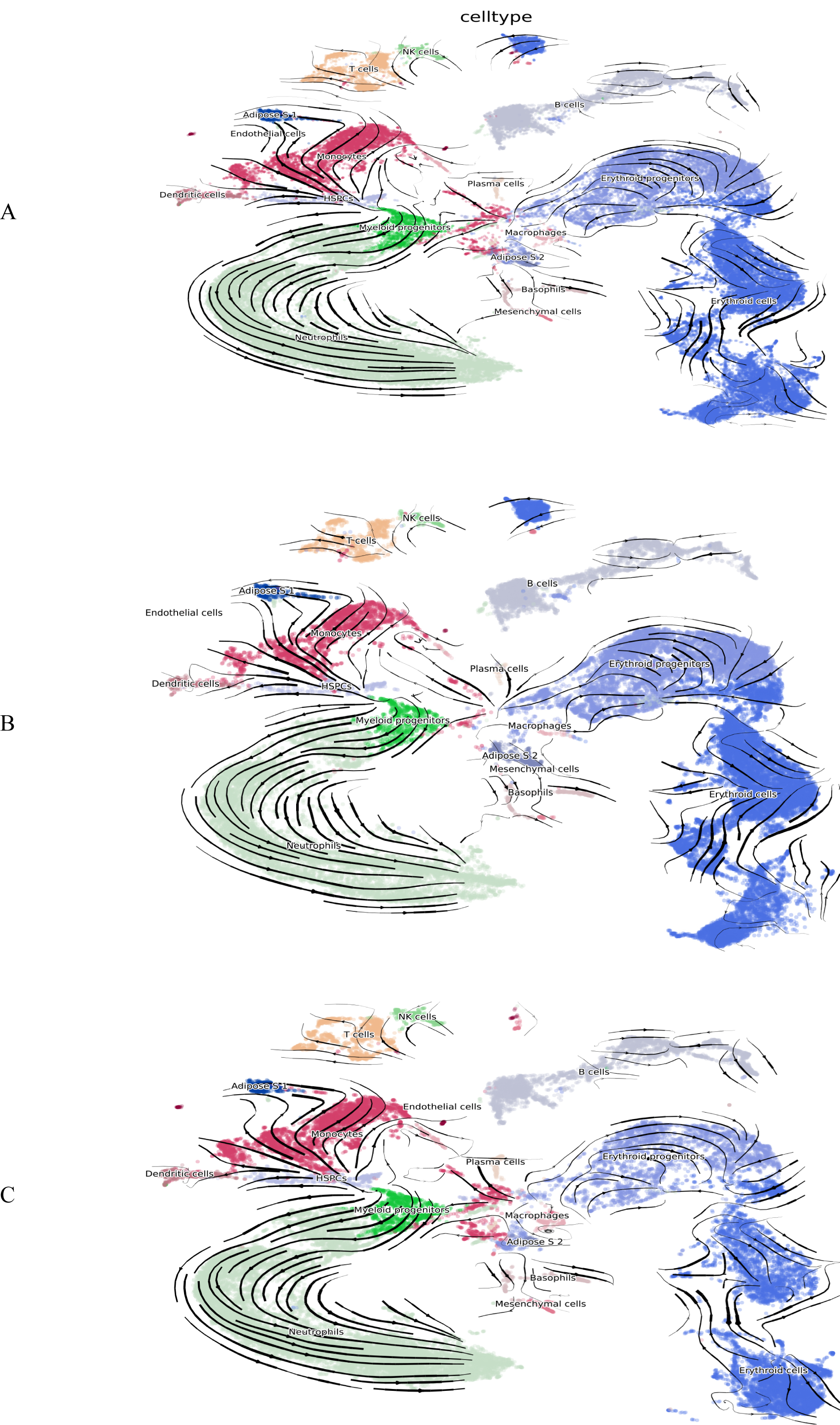

Supplement: Supplementary file 8 — High Resolution Image (TIF 20.5 MB) [file 277_2025_6605_MOESM4_ESM.tif]
